# Myeloid Protease-Activated Receptor-2 Contributes to Influenza A Virus Pathology in Mice

**DOI:** 10.3389/fimmu.2021.791017

**Published:** 2021-12-01

**Authors:** Randall C. Gunther, Vanthana Bharathi, Stephen D. Miles, Lauryn R. Tumey, Clare M. Schmedes, Kohei Tatsumi, Meagan D. Bridges, David Martinez, Stephanie A. Montgomery, Melinda A. Beck, Eric Camerer, Nigel Mackman, Silvio Antoniak

**Affiliations:** ^1^ UNC Blood Research Center, Department of Medicine, University of North Carolina at Chapel Hill, Chapel Hill, NC, United States; ^2^ UNC Blood Research Center, Department of Pathology and Laboratory Medicine, University of North Carolina at Chapel Hill, Chapel Hill, NC, United States; ^3^ UNC Lineberger Comprehensive Cancer Center, Department of Pathology and Laboratory Medicine, University of North Carolina at Chapel Hill, Chapel Hill, NC, United States; ^4^ Department of Nutrition, Gillings School of Global Public Health, School of Medicine, University of North Carolina at Chapel Hill, Chapel Hill, NC, United States; ^5^ Department of Medicine, Université de Paris, Paris Cardiovascular Research Center (PARCC), INSERM UMR 970, Paris, France; ^6^ UNC Blood Research Center, UNC Lineberger Comprehensive Cancer Center, UNC McAllister Heart Institute, Department of Pathology and Laboratory Medicine, University of North Carolina at Chapel Hill, Chapel Hill, NC, United States

**Keywords:** toll-like receptor 3, influenza A virus, innate immune response, macrophage, lung epithelial cell, protease-activated receptor 2 (PAR2), F2rl1

## Abstract

**Background:**

Innate immune responses to influenza A virus (IAV) infection are initiated in part by toll-like receptor 3 (TLR3). TLR3-dependent signaling induces an antiviral immune response and an NFκB-dependent inflammatory response. Protease-activated receptor 2 (PAR2) inhibits the antiviral response and enhances the inflammatory response. PAR2 deficiency protected mice during IAV infection. However, the PAR2 expressing cell-types contributing to IAV pathology in mice and the mechanism by which PAR2 contributes to IAV infection is unknown.

**Methods:**

IAV infection was analyzed in global (*Par2^-/-^*), myeloid (*Par2* ^fl/fl^;LysM^Cre+^) and lung epithelial cell (EpC) *Par2* deficient (*Par2^fl/fl^*;SPC^Cre+^) mice and their respective controls (*Par2* ^+/+^ and *Par2* ^fl/fl^). In addition, the effect of PAR2 activation on polyinosinic-polycytidylic acid (poly I:C) activation of TLR3 was analyzed in bone marrow-derived macrophages (BMDM). Lastly, we determined the effect of PAR2 inhibition in wild-type (WT) mice.

**Results:**

After IAV infection, *Par2^-/-^* and mice with myeloid *Par2* deficiency exhibited increased survival compared to infected controls. The improved survival was associated with reduced proinflammatory mediators and reduced cellular infiltration in bronchoalveolar lavage fluid (BALF) of *Par2^-/-^* and *Par2* ^fl/fl^;LysM^Cre+^ 3 days post infection (dpi) compared to infected control mice. Interestingly, *Par2* ^fl/fl^;SPC^Cre+^ mice showed no survival benefit compared to *Par2^fl/fl^*. *In vitro* studies showed that *Par2^-/-^* BMDM produced less IL6 and IL12p40 than *Par2* ^+/+^ BMDM after poly I:C stimulation. In addition, activation of PAR2 on *Par2* ^+/+^ BMDM increased poly I:C induction of IL6 and IL12p40 compared to poly I:C stimulation alone. Importantly, PAR2 inhibition prior to IAV infection protect WT mice.

**Conclusion:**

Global *Par2* or myeloid cell but not lung EpC *Par2* deficiency was associated with reduced BALF inflammatory markers and reduced IAV-induced mortality. Our study suggests that PAR2 may be a therapeutic target to reduce IAV pathology.

## Introduction

Influenza is a group of single-stranded RNA (ssRNA) viruses within the Orthomyxoviridae family which are responsible for over 5 million hospitalizations per year globally, occurring in young children (under the age of 2 years) and adults at the highest rates in those ≥65 years (1, 2). In particular, influenza A virus (IAV) is known for its ability to cause pandemics in the context of genetic shift, and as the long-standing major viral etiology of acute respiratory distress syndrome (ARDS) in adults (3). The ongoing coronavirus pandemic has highlighted the importance of studying the pathophysiological mechanisms underlying the course of illness and complications associated with severe respiratory viral infections.

The pathophysiology of lung inflammation and damage during influenza virus infection can be attributed to 1/virus-mediated and 2/host immune response-mediated mechanisms, with the latter including features of the innate immune response, such as neutrophil infiltration and pro-inflammatory mediator production (3). Toll-like receptors (TLRs) initiate innate immune responses by recognizing pathogen associated molecular patterns (PAMPs) (4). Double-stranded RNA (dsRNA) is a major viral PAMP generated during replication of ssRNA viruses (5, 6). TLR3 recognition of dsRNA leads to the activation of two pathways: 1/the anti-viral type-I interferon (IFN) response and 2/the NFκB pro-inflammatory response (7, 8). Importantly, TLR3 is a critical regulator of the innate immune response to IAV (7, 9). TLR3 deficiency was associated with reduced IAV-associated lung inflammation and mortality (9). Within the lung, IAV replicates primarily in epithelial cells (EpCs) and leads to damage of the EpC layer which reduces gas exchange (10, 11). However, there is evidence that replication may occur at lower levels within all cell types found in the murine lung, including alveolar macrophages (AMΦ) (11). Importantly, EpCs and AMΦ are among the first cells to response to pathogens in the lung, including IAV (12). AMΦ are one of the major sources of type-I IFN after respiratory RNA virus infections (13, 14). Moreover, AMΦ are essential in protecting against IAV infection (15, 16). However, excessive AMΦ activation contributes to IAV pathology by releasing proapoptotic factors causing direct EpC injury/death (17–19).

Protease activated receptors (PARs) are a group of four G-protein coupled receptors (PAR1-4) which are expressed broadly in humans and mice (20). For instance, PAR2 is expressed on nucleated circulating blood cells and within all organs, including the lung (20). In the lung, PAR2 is present on the surface of AMΦ and EpCs, and expression is upregulated in response to IAV (21). It was proposed that TLRs and PARs act together to detect PAMPs and infection-associated changes in protease gradients within the extracellular milieu, respectively (22). Nhu et al. (23) showed that PAR2 stimulation increased TLR3:NFκB inflammation but suppressed TLR3:type-I IFN anti-viral responses in human EpC lines *in vitro*. In addition, the authors showed that *Par2* deficiency was associated with reduced IAV-induced mortality (23).

Here, we investigate the PAR2-dependent early immune responses to IAV infection in mice. In addition, using mice with a cell-specific *Par2* deficiency, we investigated the contribution of EpC and myeloid cell expressed PAR2 to IAV-induced lung pathology. Lastly, we determined if PAR2 inhibition in wild-type (WT) mice can be a therapeutic approach to reduce IAV infection.

## Methods

### Mice

Female and male mice between 8-12 weeks of age were used in this study. *Par2* (*F2lr1*) knockout (*Par2^‐/‐^*) and their respective control (*Par2^+/+^*) mice, maintained as cousin lines, were used for this study (24). Mice carrying floxed Par2 alleles (*Par2^fl/fl^*, targeted allele name: *F2rl1* ^tm1a(EUCOMM)Wtsi^) were generated using C57Bl/6 ES cells from EUCOMM as described (25). Additional information about the *Par2^fl/fl^* mice is available at http://www.informatics.jax.org/allele/MGI:4460480. Cell-specific PAR2 deficient mice were generated by crossing female *Par2^fl/fl^* with male *Par2^fl/fl^* mice expressing Cre recombinase in a cell type‐specific manner. To generate mice with *Par2* deleted in lung EpCs we used the surfactant protein C (SPC) promoter (*Par2^fl/fl^*;SPC^Cre+^ mice) (26, 27). The *Par2* gene was deleted in the myeloid lineage (monocytes/macrophages and neutrophils) using the lysosomal M (LysM) promoter (*Par2^fl/fl^*;LysM^Cre+^) (26, 28–31). For mice with cell type-specific *Par2* deletion, littermate *Par2^fl/fl^* mice were used as controls. All mouse strains were on the C57Bl/6 background. The study was approved and performed in accordance with the guidelines of the animal care and use committee of the University of North Carolina at Chapel Hill and complies with National Institutes of Health guidelines.

### IAV Infection

Mouse-adapted influenza A/Puerto Rico/8/1934 (PR8) virus strain was propagated in 10-12 day old embryonated chicken eggs and titers were quantified by hemagglutination unit (HAU) assay (27, 32). Mice were inoculated with 0.04 HAU in 50 µl PBS administered intranasally (i.n.) as previously described (27, 32, 33). This dose results in a ~40% mortality in WT mice. Mice were given free access to feed and water while being monitored over the course of infection. Changes in body weights were recorded daily and mice were euthanized if they had ≥ 25% loss of initial body weight, as specified in our animal protocol.

### *In Vivo* PAR2 Inhibition

Eight-week old male C57BL/6J mice purchased from Jackson Laboratories (Bar Harbor, ME) were used for PAR2 inhibition studies. Thirty minutes prior to IAV infection, mice were administered i.n. 20 ng anti-mouse PAR2 antibody (SAM11, Santa Cruz Biotechnology, Dallas, TX) or IgG_2a_ control antibody (clone C1.18.4, Millipore Sigma, Burlington, MA) in 25 µl sterile normal saline to isoflurane anesthetized mice (34). Subsequently, 0.04 HAU IAV in 25 µl PBS was administered i.n. as described above. At 24 and 48 hours post-infection, mice were administered i.n. additional 20 ng and 2 µg, respectively, of SAM11 or IgG_2a_ control in 50 µl sterile normal saline.

### Bronchoalveolar Lavage Fluid Collection and Analysis

Mice were anesthetized with isoflurane and venous blood was collected from the inferior vena cava after injection of 0.2 mL sodium citrate. Mice were subsequently euthanized by cervical dislocation and bronchoalveolar lavage fluid (BALF) was collected with 3 x 900 µL ice‐cold PBS as described previously (26, 27, 32, 33). BALF samples were centrifugated and the cell free supernatant was collected (33). Cell pellets were resuspended in 200 µL PBS, and total white blood cell (WBC), neutrophil, and lymphocyte numbers were determined with an Element HT5 veterinary hematology analyzer (Heska, Loveland, CO) (26, 27, 32, 33). Lung tissue was resected, snap frozen in liquid nitrogen and stored at -80°C for further analysis. A limitation of automated cell counting for BALF cellularity is that the automated systems tends to underestimate the amount of monocytes/macrophages, especially AMΦ, in BALF preparations and potentially misrecognizes them as eosinophils (35).

### Real‐Time Polymerase Chain Reaction

Total RNA was isolated from snap frozen untreated lung or lung from lavage experiments, using the TRIzol method (Thermo Fisher Scientific) (26, 27, 32, 33). One microgram of total RNA was transcribed to complementary DNA (iScript RT Supermix Kit, Bio‐Rad Laboratories, Hercules, CA). Levels of IAV genomic RNA and IFNβ mRNA were analyzed by real‐time PCR using SSoFast Advanced Universal Supermix in a Bio‐Rad cycler (Bio‐Rad Laboratories) as described elsewhere (27, 32). Predesigned primer‐probe sets for H1N1 IAV genomic RNA and mouse IFNB1 (IFNβ) were obtained from Integrated DNA Technologies (Coralville, IA) (27, 32, 36).

### Lung Histopathology and Disease Scoring

To obtain lung tissue for histology, a subset of mice were anesthetized with isoflurane and were perfused with 2.5 mL 10U/mL heparin in PBS *via* injection into the right ventricle of the heart 7dpi as described (37). Mice were euthanized, and lungs were insufflated gently with 0.6 mL 10% phosphate-buffered formalin (37, 38). Lungs were removed and were fixed in 10% phosphate-buffered formalin, paraffin embedded, and sectioned at 4 μm. Sections (maximal airspace) of the left lung were stained with hematoxylin and eosin (H&E) (33, 38). Sections taken from similar anatomic location and were compared by a blinded pathologist for signs of lung EpC injury with focus on EpC layer disorganization, EpC layer thinning/stretching and total loss of EpC layer within the medium sized airways.

### Non-Invasive Lung Function Measurement

Global lung function was recorded on conscious mice using a Buxco whole-body plethysmography system (Data Science International, New Brighton, MN) 7dpi to quantify Penh, a measure of calculated airway resistance, EF_50_, midbreath expiratory flow, and Rpef, the rate of peak expiratory flow (39). Briefly, *Par2^+/+^* and *Par2^‐/‐^* mice were placed into individual chambers and allowed to acclimate for 20 min before a 30 min measurement window. Continuous 2-second summaries were recorded and averaged every 1 min for a total of 30 measurements per mouse (39).

### Bone Marrow-Derived Macrophages

Eight-week old male *Par2^+/+^* and *Par2^‐/‐^* mice were sacrificed by isoflurane overdose with additional cervical dislocation and femurs were excised and cleaned. Medullary cavities were flushed with ice-cold PBS and the resulting suspension was filtered through a 40-micron filter. Cells were resuspended and incubated at 37°C on 10cm cell culture petri dishes for three hours. Non-adherent cells were collected and plated on 10cm cell culture treated petri dishes at a concentration of 3 x 10^5^ cells/mL in Iscove’s Modified Dulbecco’s Media supplemented with 10% FBS (Omega Scientific, Tarzana, CA), 1% Penicillin-Streptomycin (Sigma-Aldrich, St. Louis, MO), and 50 ng/mL M-CSF (R&D Systems) with media exchange every three days. On day 7, the bone marrow-derived macrophages (BMDM) were dissociated by Trypsin-EDTA (Sigma-Aldrich) for 3 minutes and gently scraped from the plate. BMDM were seeded on 24 well or 12 well cell culture treated plates at a concentration of 2x10^5^ cells/mL in DMEM/F12 supplemented with 10% FBS, 100 mM L-glutamine, and 1% Penicillin-Streptomycin 36 hours prior to stimulation. Media was exchanged and BMDM were stimulated with 5 µg/mL polyinosinic-polycytidylic acid (poly I:C, Tocris, Minneapolis, MN) and/or 200 µM PAR2 agonist peptide (PAR2 AP, SLIGRL‐NH_2_, R&D Systems).

### ELISA

Protein levels of TNFα, MCP1, CXCL1, IL1β, IL6, and IL12p40 in BALF and BMDM conditioned media was analyzed by ELISA (Duo-Set, R&D Systems, Minneapolis, MN) (26, 27, 32, 33, 40).

### Statistics

GraphPad Prism 9.2 (GraphPad Software Inc, San Diego, CA) was used for statistical analysis. Data are represented as mean ± standard error of the mean (SEM). The two‐tailed Student *t* test was used for two‐group comparison of normally distributed data. For multiple‐group comparison, normally distributed data were analyzed by two‐way ANOVA test and were Bonferroni‐corrected for repeated measure over time. Survival rates were analyzed by Kaplan–Meier analysis and the log‐rank test was applied to compare the survival distribution between the two groups. *P* value ≤ 0.05 was regarded as significant.

## Results

### *Par2* Deficiency Is Associated With Reduced IAV-Induced Mortality

**Figure 1:**
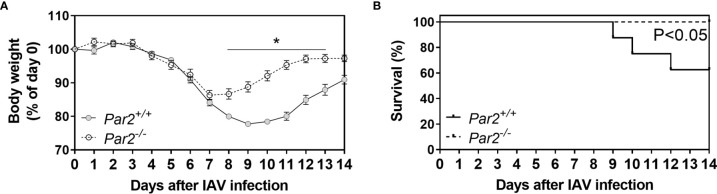
PAR2 deficiency is associated with improved survival after IAV infection. *Par2^+/+^* and *Par2^-/-^* mice were infected with 0.04 HAU IAV and changes in body weights were recorded daily for 14 days **(A)**. Overall survival **(B)** was observed as well as calculated from body weights for 14 days. A weight loss of ≥25% of the initial body weight was set as compassionate humane endpoint as specified in the animal protocol. Body weights before infection (day 0) was set to 100%. Data (mean ± SEM) and calculated survival were analyzed by two-way ANOVA **(A)** and log-rank test **(B)**. *P < 0.05.

Mice were monitored daily for 14 days for weight loss following IAV infection. Weight loss ≥25% or actual death were criteria for mortality. Body weight curves were constructed showing daily weights of mice remaining that had not met mortality criteria. (**Figure 1A**). After infection, mice of both genotypes exhibited similar body weight changes up to 7 days post infection (dpi) (**Figure 1A**). However, *Par2^-/-^* mice exhibited improved body weight recovery compared to *Par2^+/+^* mice starting 8dpi. The calculated Kaplan Meier survival curves constructed for mice over the course of the infection showed that *Par2^-/-^* mice had significantly improved survival compared to infected *Par2* ^+/+^ mice 14dpi (P<0.05) (**Figure 1B**).

### PAR2 Regulates Cytokine and Neutrophil Accumulation in the Airspace After AV Infection

**Figure 2:**
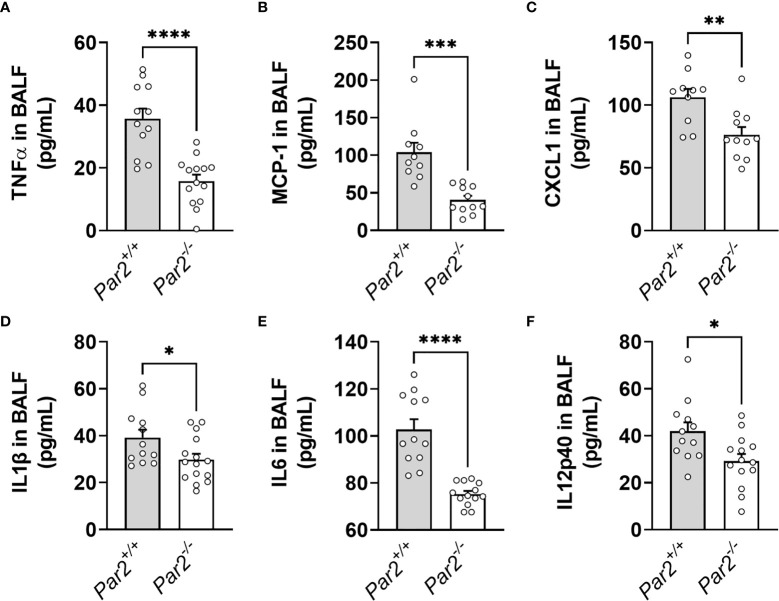
PAR2 deficiency results in reduced inflammation in the airspace after IAV infection. *Par2^+/+^* and *Par2^-/-^* mice were infected with 0.04 HAU IAV and bronchoalveolar lavage fluid (BALF) was analyzed for TNFα **(A)**, MCP1 **(B)**, CXCL1 **(C)**, IL1β **(D)**, IL6 **(E)** and IL12p40 **(F)** protein levels 3 days after infection by ELISA. Data (mean ± SEM) was analyzed by Student t test. *P < 0.05, **P < 0.01, ***P < 0.005, ****P < 0.001.

**Figure 3:**
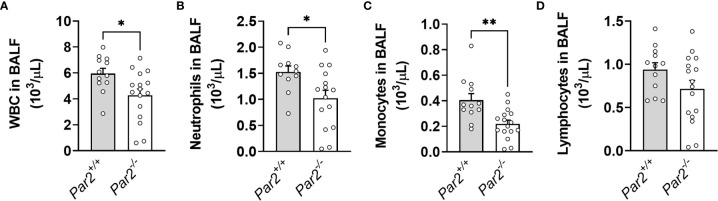
PAR2 deficiency results in reduced cellular inflammation in the airspace after IAV infection. *Par2^+/+^* and *Par2^-/-^* mice were infected with 0.04 HAU IAV and bronchoalveolar lavage fluid (BALF) cellularity was analyzed by automated cell counter for total white blood cell (WBC) **(A)**, neutrophil **(B)**, monocytes **(C)** and lymphocyte **(D)** numbers 3 days after infection. Data (mean ± SEM) was analyzed by Student t test. *P < 0.05, **P < 0.01.

Severe IAV infection provokes monocyte/macrophage and neutrophil infiltration that can drive IAV-induced pathology (17–19). Importantly, neutrophils have been implicated in a feed-forward pathogenic program in IAV infection (41). To evaluate the role of PAR2 in early inflammatory responses in the airspace after IAV infection, BALF of *Par2^+/+^* and *Par2^-/-^* mice was collected 3dpi and assayed for proinflammatory cytokines/chemokines and infiltrating immune cell numbers. As expected, *Par2^-/-^* mice had significantly reduced levels of a subset of proinflammatory mediators, including TNFα, MCP1, CXCL1, IL1β, IL6, and IL12p40 compared to *Par2^+/+^* mice (**Figures 2A–F**). Moreover, decreased total white blood cell, neutrophil and monocyte numbers were observed in BALF of *Par2^-/-^* mice compared to BALF of *Par2* ^+/+^ mice at 3dpi (**Figures 3A–C**). There were no significant differences detected in levels of lymphocytes in the BALF of the two genotypes at 3dpi (**Figure 3D**). In addition, *Par2* ^-/-^ BALF exhibited reduced eosinophil numbers compared to *Par2* ^+/+^ mice BALF at 3dpi (**Supplement Figure 1**). Some of these cells may be AMΦ because the automated cell counter cannot easily distinguish these cell types (35). However, at 7dpi there were, with exception for IL6, similar BALF inflammatory mediator levels in the two genotypes (**Supplement Figure 2**). Importantly, while TNFα, MCP1, CXCL1 and IL12p40 BALF levels were no longer different between the two genotypes at 7dpi, BALF of *Par2* ^+/+^ mice still exhibited increased cellularity with significantly higher total WBC, neutrophil, and monocyte numbers compared to *Par2^-/-^* mice BALF 7dpi (**Supplement Figure 3**). Moreover, lymphocytes and eosinophils numbers in BALF were similar between the two genotypes at 7dpi (**Supplement Figure 3**).

### *Par2* Deficiency Is Associated Increased IFNβ Expression and Reduced IAV Genome Levels in the Lung

**Figure 4:**
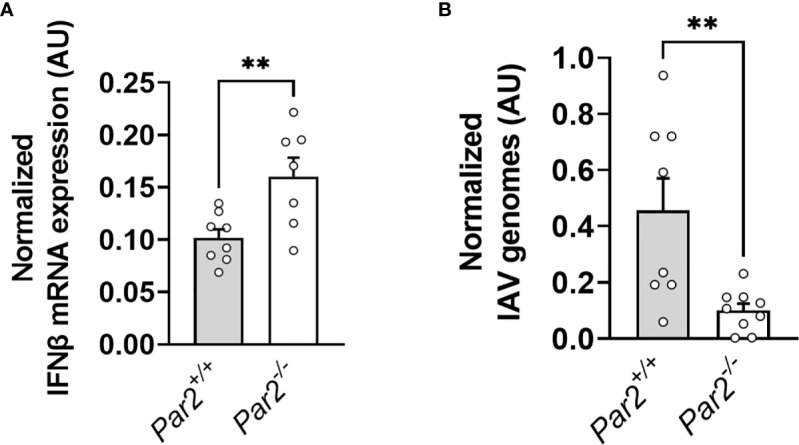
PAR2 deficiency results in IFNβ expression and reduced virus load in the lung after IAV infection. *Par2^+/+^* and *Par2^-/-^* mice were infected with 0.04 HAU IAV and IFNβ mRNA expression **(A)** and IAV genomes **(B)** in the lungs were analyzed by RT-PCR 3 days after infection. Data (mean ± SEM) was analyzed by Student t test. **P < 0.01.

Type-I IFN signaling was shown to restrict IAV replication and pathologic inflammatory immune responses in the IAV infected lung (42). We and others showed that *Par2* deficiency was associated with increased IFNβ expression *in vivo* and *in vitro* (23, 43). In addition, we linked PAR2 expression and activation to increased Coxsackievirus B3 replication *in vitro* (43). To analyze the effect of PAR2 expression on antiviral IFNβ expression and IAV replication in infected lungs, RNA was isolated and IFNβ mRNA as well as IAV genomes measured in lungs of *Par2^+/+^* and *Par2^-/-^* mice 3dpi. Importantly, infected *Par2^-/-^* mice lungs exhibited increased IFNβ mRNA expression compared to infected *Par2^+/+^* mice lungs 3dpi (**Figure 4A**). In line with increased antiviral response, *Par2* deficiency was associated with reduced IAV genome levels in the lung compared to *Par2^+/+^* mice lungs 3dpi (**Figure 4B**).

### PAR2 Contributes to Lung Epithelial Cell Loss During IAV Infection

**Figure 5:**
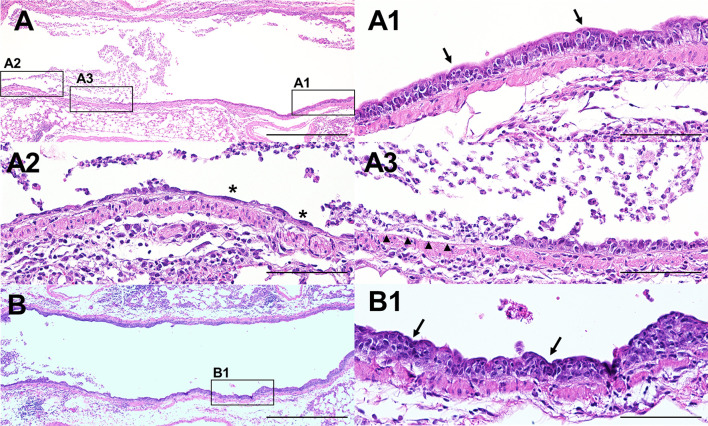
PAR2 expression is associated with increased lung epithelial cell injury after IAV infection. *Par2^+/+^* and *Par2^-/-^* mice were infected with 0.04 HAU IAV and lung sections were cut for maximal airspace and stained with H&E staining 7 days after infection. **(A)** Representative overview of a section of the main left bronchi (maximal airspace) of an IAV-infected *Par2^+/+^* mouse. Overall, *Par2^+/+^* mice exhibited more signs of lung epithelial cell injury including disorganization of the epithelial cell layer (**A1**, arrows), epithelial cell thinning/stretching (**A2**, asterisks) and total loss of epithelial cells/denudation (**A3**, arrow heads). Representative overview of a section of the main left bronchi of an IAV-infected *Par2^-/-^* mouse **(B)** shows a less severe lung epithelial cell injury phenotype with apparent signs of epithelial cell disorganization **(B1**, arrows). Size bar=1mm **(A, B)** and size bar=200µm **(A1–A3, B1)**.

IAV primarily infects and replicates in lung epithelium which results in loss of alveolar and bronchial EpCs up to 7dpi. Repair of the EpC layer begins after day 7 when surviving mice start regaining body weight (44). Importantly, loss of more than 10% of alveolar EpCs is correlated with increased mortality in IAV-infected mice (45). To analyze IAV-induced lung EpC injury formalin-fixed and paraffin-embedded lung sections of IAV-infected *Par2^+/+^* and *Par2^-/-^* mice (7dpi) were cut to maximal airspace and stained with H&E. The most striking difference between *Par2^+/+^* (**Figure 5A**) and *Par2^-/-^* (**Figure 5B**) mice was that infected *Par2^+/+^* mice exhibited more signs of lung EpC injury compared with *Par2^-/-^* mice. This included more severe disorganized lung EpC layer (**Figure 5A1**) indicating concurrent cell damage and regeneration, EpC stretching/thinning (**Figure 5A2**) and total EpC loss (denudation, **Figure 5A3**), accumulation of neutrophils and cellular debris in the airway lumen compared to infected *Par2^-/-^* mice (**Figure 5B**) which showed only infection-induced disorganization of the EpC layer (**Figure 5B1**). The obvious changes in the lung epithelial histology suggest that PAR2-dependent inflammation during IAV infection leads to more pronounced lung EpC injury which may explain the delayed body weight recovery and the increased mortality in *Par2^+/+^* mice as shown in **Figure 1**.

### *Par2* Deficiency Is Associated With Improved Lung Function After IAV Infection

**Figure 6:**
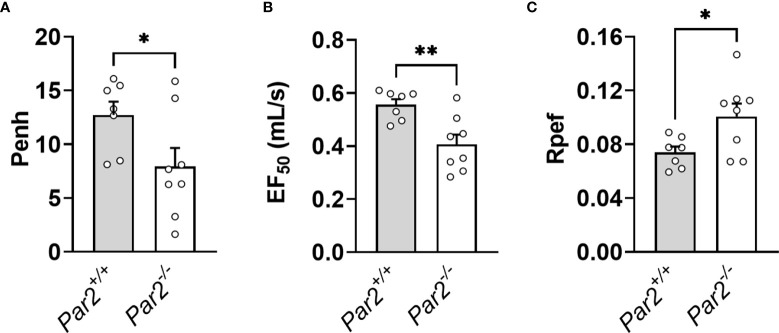
PAR2 deficiency was associated with less severe lung dysfunction after IAV infection. *Par2^+/+^* and *Par2^-/-^* mice were infected with 0.04 HAU IAV and lung function was recorded using a Buxco whole-body plethysmography system 7 days after infection for Penh, a measure of calculated airway resistance **(A)**, EF_50_, midbreath expiratory flow **(B)**, and Rpef, the rate of peak expiratory flow **(C)**. Data (mean ± SEM) was analyzed by Student t test. *P < 0.05. **P < 0.01.

IAV infection-associated pathology results in impaired lung function with increased airways resistance, increased exhalation force and reduced peak exhalation flow (39). To measure the global lung function, *Par2^+/+^* and *Par2^-/-^* mice (7dpi) subjected to Buxco whole-body plethysmography system (39). Enhanced pause (Penh) is a calculated measure of airway resistance that is associated with airway denudation, airway debris and immune cell accumulation in the airway following IAV infection (39). The 50% exhalation force (EF_50_) measures the exhalation force midbreath, which increases as breathing becomes more difficult. Finally, the ratio of peak expiratory flow (Rpef) is the time to peak expiratory flow and has been associated with wheezing following infection (39). All three metrics have been shown to change significantly following IAV infection, with Penh and EF_50_ increasing following infection and Rpef decreasing (39). In line with our findings, *Par2^+/+^* mice exhibited increased Penh (**Figure 6A**), increased EF_50_ (**Figure 6B**) but reduced Rpef (**Figure 6C**) compared to *Par2^-/-^* mice 7dpi. Combined, these measurements show that *Par2* deficiency was associated with improved lung function after IAV infection.

### The Effect of *Par2* Deletion in Either Epithelial or Myeloid Cells on IAV Mortality

**Figure 7:**
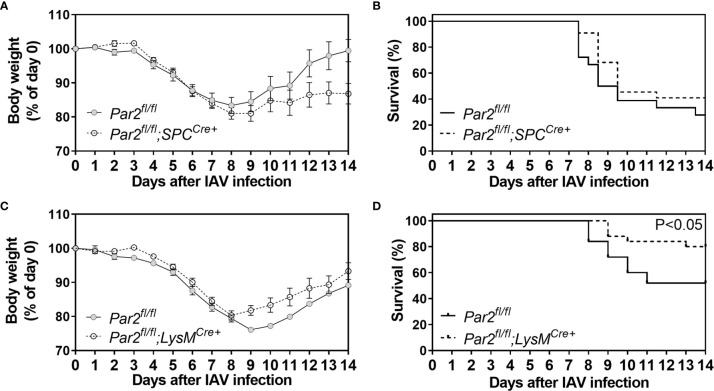
PAR2 deficiency on myeloid cells was associated with improved survival after IAV infection. Mice with *Par2* deficiency in lung epithelial cells (*Par2^fl/fl^*,SPC^Cre+^) or myeloid cells (*Par2^fl/fl^*,LysM^Cre+^) and their littermates controls (*Par2^fl/fl^)* were infected with 0.04 HAU IAV. Changes in body weights were recorded daily for 14 days after infection **(A, C)**. Overall survival **(B, D)** was observed as well as calculated from body weights for 14 days. A weight loss of ≥25% of the initial body weight was set as compassionate humane endpoint as specified in the animal protocol. Body weights before infection (day 0) were set to 100%. Data (mean ± SEM) and calculated survival were analyzed by two-way ANOVA **(A, C)** and log-rank test **(B, D)**.

A previous study using cultured lung EpCs suggested that PAR2 on lung EpCs contributes to IAV pathology (23). In addition, other studies implied a major role of PAR2 on myeloid cells for immune response modulation (28, 46). Here, we investigated the effect of cell-specific *Par2* deletion in lung EpCs (*Par2^fl/fl^*;SPC^Cre+^) or myeloid cells (*Par2^fl/fl^*;LysM^Cre+^) on IAV infection. Body weights were monitored daily after IAV infection and a weight loss ≥25% or actual death were criteria for a mortality event. Body weight curves were constructed showing daily weights of mice remaining who had not met mortality criteria (**Figures 7A, C**). *Par2^fl/fl^*;SPC^Cre+^ mice had slightly reduced body weights 7-14dpi compared to controls (**Figure 7A**) but the differences did not reach statistical significance. Moreover, Kaplan-Meier survival analysis showed no significant differences in surviving mice throughout the course of infection for *Par2^fl/fl^*;SPC^Cre+^ mice compared to their controls (*Par2^fl/fl^*) (**Figure 7B**). *Par2^fl/fl^*;LysM^Cre+^ mice showed a slightly improved total body weight recovery than control mice (**Figure 7C**) but again this difference did not reached statistical significance. However, Kaplan-Meier survival curves showed that *Par2^fl/fl^*;LysM^Cre+^ mice had significantly reduced IAV mortality over the course of the observational period of 14 days when compared to control *Par2^fl/fl^* mice (**Figure 7D**).

### Myeloid PAR2 Regulates Proinflammatory Response and Neutrophil Accumulation in IAV-Infected Mouse Lungs

**Figure 8:**
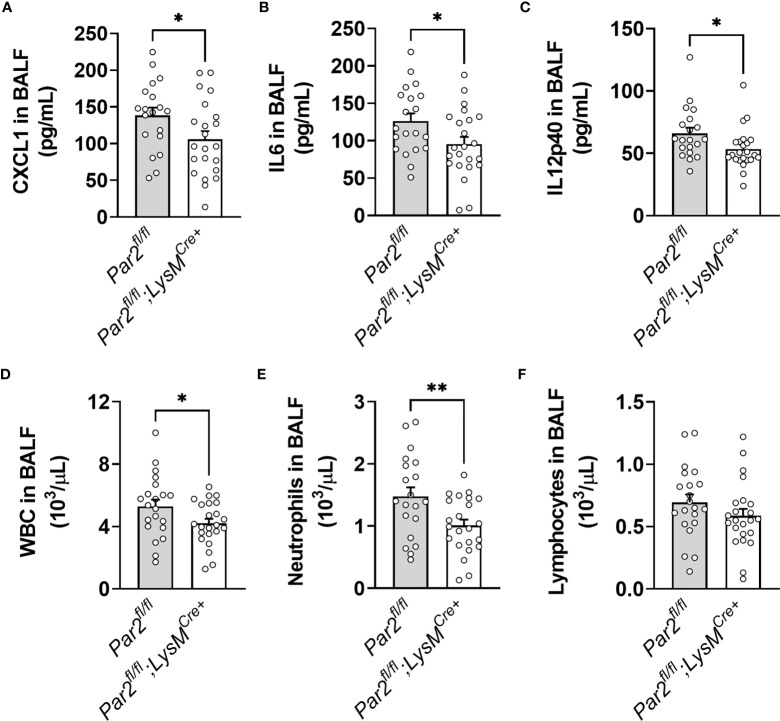
Myeloid cell *Par2* deficiency results in reduced inflammation in the airspace after IAV infection. *Par2^fl/fl^*,LysM^Cre+^ and their littermate controls *Par2^fl/fl^* mice were infected with 0.04 HAU IAV and bronchoalveolar lavage fluid (BALF) was analyzed for CXCL1 **(A)**, IL6 **(B)** and IL12p40 **(C)** levels 3 days after infection by ELISA. BALF cellularity was analyzed by automated cell counter for total white blood cell (WBC) **(D)**, neutrophil **(E)** and lymphocyte **(F)** numbers 3 days after infection. Data (mean ± SEM) was analyzed by Student t test. *P < 0.05, **P < 0.01.

Since only *Par2^fl/fl^*;LysM^Cre+^ mice exhibited a survival benefit after IAV infection compared to *Par2^fl/fl^*;SPC^Cre+^ mice and *Par2^fl/fl^* mice, we focused the subsequently analysis on *Par2^fl/fl^*;LysM^Cre+^ mice and compared them to *Par2^fl/fl^* control mice. BALF of *Par2^fl/fl^*;LysM^Cre+^ mice and their *Par2^fl/fl^* controls were analyzed 3dpi and assayed for proinflammatory mediators and immune cell numbers. In line with the improved survival, *Par2^fl/fl^*;LysM^Cre+^ mice had significantly reduced levels of CXCL1, IL6, and IL12p40 in BALF compared to littermate *Par2^fl/fl^* controls 3dpi (**Figures 8A–C**). Likewise, decreased total white blood cells and neutrophils numbers were measured in BALF of *Par2^fl/fl^*;LysM^Cre+^ compared to control *Par2^fl/fl^* mice 3dpi (**Figures 8D, E**). There were no significant differences detected in levels of BALF lymphocytes (**Figure 8F**).

### *Par2* Deficiency in Myeloid Cells Results in Increased IFNβ Expression and Reduced IAV Genome Levels in the Lung

**Figure 9:**
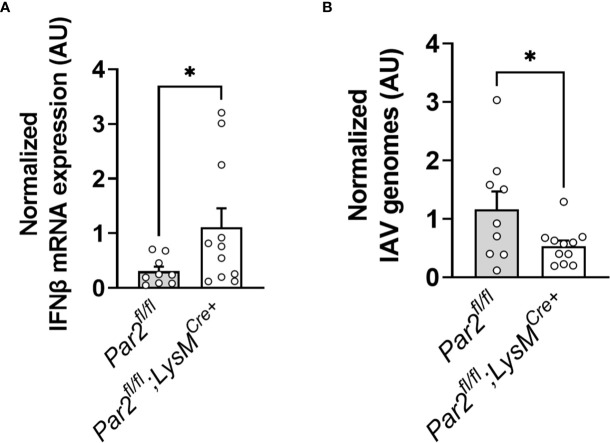
Myeloid *Par2* deficiency was associated with increased IFNβ expression but reduced H1N1 IAV virus genomes levels in the IAV infected lung. *Par2^fl/fl^*,LysM^Cre+^ and their littermate controls *Par2^fl/fl^* mice were infected with 0.04 HAU IAV and IFNβ mRNA expression **(A)** and IAV genome levels **(B)** in the lungs were analyzed by RT-PCR 3 days after infection. Data (mean ± SEM) was analyzed by Student t test. *P < 0.05.

Macrophages are able to restrict/abort IAV replication after infection (47). However, reduced type-I IFN signaling on macrophages renders the cells more susceptible for productive IAV replication (47). To analyze the effect of *Par2* deficiency in myeloid cells on lung IFNβ expression and overall IAV replication, *Par2^fl/fl^* and *Par2^fl/fl^*;LysM^Cre+^ were infected with IAV and total RNA from lungs isolated 3dpi. Importantly, myeloid cell *Par2* deficient mice had higher IFNβ expression in the lung compared to the infected *Par2^fl/fl^* littermates (**Figure 9A**). In line with the increased antiviral response in *Par2^fl/fl^*;LysM^Cre+^, the mice with myeloid *Par2* deficiency had also reduced IAV genome levels in the lung compared to the infected *Par2^fl/f^* littermates 3dpi (**Figure 9B**).

### PAR2 Activation Augments Poly I:C Induction of IL6 and IL12p40 Expression in Bone Marrow-Derived Macrophages

**Figure 10:**
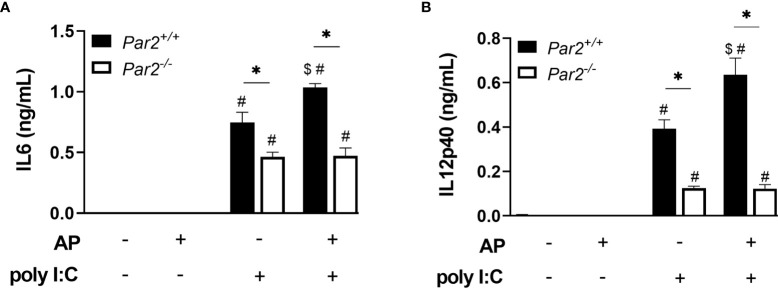
PAR2 activation of macrophages increases IL6 and IL12p40 expression during TLR3 stimulation. Bone-marrow derived macrophages were stimulated with poly I:C (5µg/mL) and/or PAR2 agonist (AP, 200µM) under serum-free conditions. IL6 **(A)** and IL12p40 **(B)** levels were measured in the culture media 24 hrs after stimulation by ELISAs. Data (mean ± SEM) was analyzed by 2-Way ANOVA. *P < 0.05, *# vs*. unstimulated control within the same genotype, ^$^P < 0.05 *vs*. poly I:C alone within the same genotype.

BMDM form *Par2^+/+^* and *Par2^-/-^* mice were cultured *in vitro* to further evaluate the role of myeloid (macrophage) cell PAR2 in coordinating the inflammatory response to RNA viruses including IAV. The TLR3 agonist poly I:C was used to mimic virus-like stimulation *in vitro*. Poly I:C induced IL6 or IL12p40 expression in both genotypes. However, *Par2^+/+^* BMDM produced more IL6 and IL12p40 in response to poly I:C when compared to *Par2^-/-^* BMDM (**Figures 10A, B**). PAR2 stimulation alone did not significantly increased the IL6 or IL12p40 levels over the baseline. Importantly, *Par2^+/+^* BMDM costimulated with PAR2 AP and poly I:C express significantly higher levels of IL6 or IL12p40 compared to poly I:C alone. As expected, the PAR2 AP did not elicit an increased response in *Par2* ^-/-^ BMDM treated with poly I:C (**Figure 10**).

### PAR2 Inhibition Results in Decreased Cytokine Production in the MouseLung After IAV Infection

**Figure 11:**
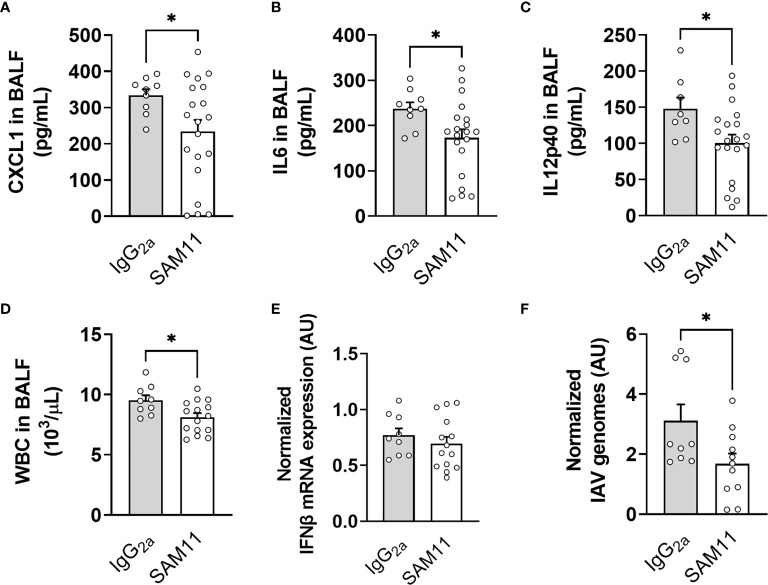
PAR2 inhibition prior to infection resulted in reduced inflammation in the lung after IAV infection. WT mice were treated intranasally with anti-mouse PAR2 antibody (SAM11) prior to infection with 0.04 HAU IAV and then daily for 3 days (see *Methods* for additional information). Bronchoalveolar lavage fluid (BALF) was collected at day 3 post IAV infection and analyzed for CXCL1 **(A)**, IL6 **(B)** and IL12p40 **(C)** levels by ELISA. White blood cells (WBC) numbers **(D)** were analyzed by automated cell counter. Lung IFNβ mRNA expression **(E)** and IAV genome levels **(F)** were analyzed by RT-PCR 3 days after infection. Data (mean ± SEM) was analyzed by Student t test. *P < 0.05.

To evaluate the potential of intranasal PAR2 antagonist treatment to reduce pathologic inflammation in the lung after IAV infection, WT mice were treated with an inhibitory PAR2 antibody (SAM11) or control IgG_2a_ prior and during infection with IAV. BALF of mice treated with SAM11 or IgG_2a_ control was collected at 3dpi and assayed for proinflammatory mediators and cellular infiltrate. SAM11 treatment resulted in significantly reduced levels of CXCL1, IL-6, and IL-12p40 in BALF compared control IgG_2a_-treated mice 3dpi (**Figures 11A–C**). In line with this, SAM11-treated mice had decreased total white blood cells in BALF compared to IgG_2a_-treated controls (**Figure 11D**). While SAM11 treatment did not change the expression of IFNβ it resulted in reduced overall IAV genome levels in the lung compared to IgG_2a_ treated mice 3dpi (**Figures 11E, F**).

## Discussion

In this study, we showed that PAR2 contributes to IAV infection-induced mortality in mice. In addition, we found that PAR2 contributes to increased cytokine expression and immune cell infiltration into the air space (BALF) leading to more pronounced global lung dysfunction in mice after IAV infection. Using mice with cell-specific deletion of *Par2*, we observed that myeloid-expressed PAR2, but not lung EpC PAR2 contributed to IAV pathology. Importantly, prophylactic PAR2 inhibition using an anti-mouse PAR2 antibody reduced IAV progression in mice.

**Figure 12:**
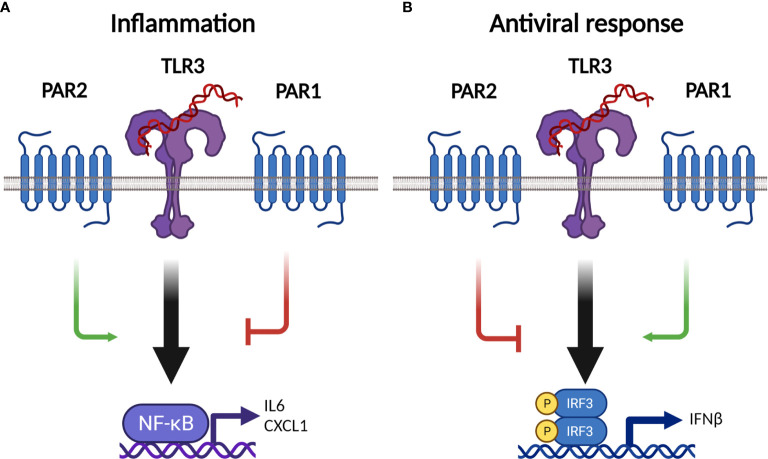
Proposed model how PAR2 and PAR1 modulates TLR3 responses during influenza A virus infection. **(A)** PAR2 enhances and PAR1 dampens TLR3-NFκB-dependent expression of inflammatory markers in the bronchoalveolar lavage fluid after influenza A virus (IAV) infection. **(B)** With regard to the anti-viral TLR3-IFNβ response, PAR2 dampens and PAR1 enhances IFNβ expression in the lung after IAV infection. See main text for additional information. CXCL1, C-X-C Motif Chemokine Ligand 1; IFNβ, interferon-beta; IL6, interleukin 6; PAR, protease activated receptor; TLR3, toll-like receptor 3. Figure created on Biorender.com.

Based on our studies of PARs in ssRNA virus infections, as well by others, we proposed a model in which PAR2 enhances TLR3-NFκB inflammation but reduces TLR3-type-I IFN responses. In contrast, PAR1 reduces TLR3-NFκB inflammation but enhances TLR3-IFNβ responses (**Figure 12**) (8, 23, 36, 43, 48). In line with this proposed receptor interaction, we have recently shown that the absence of PAR1 leads to increased proinflammatory CXCL1 expression and increased BALF neutrophil numbers which were associated with higher mortality compared to WT mice (26).

There are conflicting data about the role of PAR2 in IAV infection in mice and cells *in vitro*. Our data presented here are consistent with the observation by Vogel’s group that *Par2* deficiency was associated with improved survival after IAV infection (23). Importantly, we used a different line of *Par2*-deficient mice (24, 49) compared with Nhu et al. (23) but made similar observations after IAV infection supporting a role of PAR2 in IAV pathology. In contrast to our and Vogel’s findings, Riteau’s group showed that *Par2^-/-^* mice exhibited increased mortality after IAV infection with either 30 plaque-forming units (pfu) or 60 pfu (50). Interestingly, using this dose the authors did not induce any body weight changes or death in WT mice (50). Moreover, studies using the specific PAR2 AP (SLIGRL-NH_2_) suggested that PAR2 activation mediates a protective mechanism in IAV infection in mice and in *in vitro* cell culture system (50–55). However, SLIGRL-NH_2_ has been reported to inhibit IAV infection in mice and *in vitro* independently of PAR2 (52, 55).

In general, IAV infection-mediated pathology is caused by a lack of adequate innate antiviral immune responses causing virus induced injury which can be exacerbated by an excessive proinflammatory response (3, 41). Importantly, the overreacting host’s immune response appears to contribute to the morbidity and mortality after IAV infection (41). For instance, TLR3-deficient mice exhibited improved survival associated with reduced lung inflammation while having an increased virus load after IAV infection compared to WT mice (56). Nhu et al. showed that PAR2 activation increased NFκB responses but reduced type-I IFN responses during TLR3 stimulation of lung EpCs (23). By extrapolation of their *in vitro* observations, the authors suggested that PAR2 activation on lung EpCs would contribute to IAV pathology *in vivo* (23). We did not observe a lung EpC PAR2-dependent mortality phenotype in our IAV infection model. However, body weight recovery seemed different between *Par2^fl/fl^* and *Par2^fl/fl^*;SPC^Cre+^ mice suggesting a protective role for EpC PAR2 in maintaining barrier function (57). The mouse-adapted PR8 IAV strain is highly pathogenic and might overwhelm any PAR2-dependent effects in EpCs *in vivo*. However, we found that myeloid PAR2 expressing cells increased NFκB-associated lung inflammation in PR8 IAV infected mice. Moreover, we found that PAR2 expression further reduced IFNβ expression in the lung 3dpi. Using BMDM, we confirmed that PAR2 expression and activation increased the release of the two NFκB-dependent cytokines IL6 and IL12p40 during TLR3 stimulation *in vitro*. Moreover, neutrophils can play a protective as well as detrimental role in IAV infection, and PAR2 stimulation can increase neutrophil activity (53). While neutrophil depletion led to increased IAV infection, an overactivation and increased neutrophil recruitment to the lung after IAV infection was shown to be associated with increased IAV-induced pathology and death (26, 41, 58). Although neutrophils are of myeloid lineage and targets of LysM^Cre^-activity (30), we did not specifically address whether PAR2 on neutrophils plays a role on IAV progression in this study.

Together with past studies using other viruses, including IAV and Coxsackievirus B3, or sterile virus-like stimulation with poly I:C, this study indicates that PAR2 expression and activation contributes to viral infection-associated pathology by enhancing proinflammatory TLR3-NFκB responses and reducing antiviral TLR3-IFNβ responses as first suggested first by Nhu et al. (23, 43, 59–61). How does PAR2 mediate its effect on TLR3 signaling? As previous demonstrated by Vogel’s group (62), we showed that PAR2 can be immunoprecipitated with TLR4 and TLR3 (43). It is not clear if the physical interaction alone can explain the observed phenotype. While PAR2 has no immediate effect on IFNβ signaling (within the first 15 min) (23) but it reduces IFNβ signaling at later stages (past 180 min) (61). Whether PAR2 activation directly dampens IFNβ-dependent STAT1 activation, increases STAT1 dephosphorylation or reduces interferon-α/β receptor surface expression is unclear. Of note, PAR2-dependent reduction of the TLR3-IFNβ pathway activation was linked to PAR2-dependent activity of the tyrosine phosphatase SH2 domain-containing protein tyrosine phosphatase-2 (SHP-2, protein tyrosine phosphatases [PTP] 11) (61). In line with our findings, SHP-2 activity was shown to be important for efficient NFκB activation (63). Moreover, *in vivo* PAR2 AP stimulation of murine urinary bladders increased the expression of the dual specificity phosphatase 1 (DUSP1, mitogen-activated protein kinase [MAPK] phosphatase 1) (64) which is known to inactivate the MAPKs JNK and p38. Interestingly, DUSP1 expression/activity reduces TLR3-mediated IFNβ expression in macrophages by reducing JNK-dependent IFNβ gene transcription and reducing p38-dependent IFNβ mRNA stability (65).

PAR2 can be activated by a variety of proteases including trypsin, tryptase, neutrophil elastase, different membrane-bound proteases, the tissue factor (TF)/FVIIa complex, or FXa alone. We showed that IAV infection increases lung EpC TF expression which leads to IAV-associated local activation of coagulation (27). This suggests that the TF/FVIIa complex is formed and FXa is generated locally during IAV infection in the lung which could in turn lead to PAR2 activation. Immune cell expressed proteases are also present in the lung during IAV infection and pulmonary expressed membrane-bound proteases including transmembrane protease serine type 2 (TMPRSS2), matriptase or human airway trypsin-like protease are known to activate PAR2 (66–68). Interestingly, TMPRSS2 deficiency was shown to reduce inflammatory responses to intranasal poly I:C (69). In an IAV-induced myocarditis model, local trypsin expression was associated with increased cardiac pathology (70, 71). However, the authors did not link the increased trypsin expression to increased PAR2 signaling. Importantly, PAR2-activating proteases are involved in the proteolytic activation of IAV (72, 73). In line with this, serine protease inhibitors, including aprotinin (74) and camostat mesylate (75), were shown to directly reduce IAV infectivity and might also reduce protease-dependent PAR2 activation during IAV infection.

Antiviral treatments for influenza virus infections are limited (76, 77). We show that PAR2 inhibition prior to infection not only reduced IAV virus genome levels in the lung 3dpi but also reduced cytokine/chemokine and cellular inflammation in the BALF compared to control IgG treated mice. In support to our findings, PAR2 inhibition reduced immune cell infiltration into the lung/airspace of respiratory syncytial virus infected mice (78). These findings suggest that PAR2 might be a therapeutic target in reducing respiratory viral infection, including pandemic coronavirus infections (79–81).

In conclusion, we linked myeloid cell PAR2 to the IAV pathology in mice. PAR2 not only reduces antiviral type-I IFN responses but also enhances NFκB-dependent inflammation in the lung of IAV infected mice resulting in increased BALF cellularity, which was associated with increased lung EpC injury, overall more pronounced global lung dysfunction and higher mortality. Moreover, we show that PAR2-directed therapeutics have the potential not only to enhance antiviral immune responses to IAV but also to reduce host-driven pathologic lung inflammation.

## Data Availability Statement

The raw data supporting the conclusions of this article will be made available by the corresponding author, without undue reservation.

## Ethics Statement

The animal study was reviewed, approved and performed in accordance with the guidelines of the animal care and use committee of the University of North Carolina at Chapel Hill and complies with National Institutes of Health guidelines.

## Author Contributions

RG, VB, SDM, LT, CS, KT, MDB, and DM conducted experiments. SAM performed the blinded histological evaluation of the lung sections and provided additional data interpretation. RG, NM, and SA interpreted the data and wrote the manuscript. MB and EC provided essential materials and edited the manuscript. NM and SA provided funding. SA designed and overviewed the study. All authors contributed to the article and approved the submitted version.

## Funding

The presented study was supported by grants from the NHLBI to NM (1R35HL155657) and SA (1R01HL142799).

## Conflict of Interest

The authors declare that the research was conducted in the absence of any commercial or financial relationships that could be construed as a potential conflict of interest.

## Publisher’s Note

All claims expressed in this article are solely those of the authors and do not necessarily represent those of their affiliated organizations, or those of the publisher, the editors and the reviewers. Any product that may be evaluated in this article, or claim that may be made by its manufacturer, is not guaranteed or endorsed by the publisher.

## Supplementary Material

Supplement Figure 1 PAR2 deficiency was associated with reduced eosinophil numbers in the airspace 3 days after IAV infection. *Par2* ^+/+^ and *Par2* ^-/-^ mice were infected with 0.04 HAU IAV and eosinophil numbers in bronchoalveolar lavage fluid (BALF) was analyzed by automated cell counter 3 days after infection. Data (mean ± SEM) was analyzed by Student t test. *P < 0.05.

Supplement Figure 2 Cytokine levels in airspace of *Par2* ^+/+^ and *Par2* ^-/-^ mice 7 days after influenza A virus infection. *Par2* ^+/+^ and *Par2* ^-/-^ mice were infected with 0.04 HAU IAV and bronchoalveolar lavage fluid (BALF) was analyzed for TNFα **(A)**, MCP-1 **(B)**, CXCL1 **(C)**, IL6 **(D)** and IL12p40 **(E)** protein levels 7 days after infection by ELISA. Data (mean ± SEM) was analyzed by Student t test. ***P < 0.005.

Supplement Figure 3 PAR2 deficiency was associated with reduced immune cell numbers in the airspace 7 days after influenza A virus infection. *Par2* ^+/+^ and *Par2* ^-/-^ mice were infected with 0.04 HAU IAV and bronchoalveolar lavage fluid (BALF) cellularity was analyzed by automated cell counter for total white blood cell (WBC) **(A)**, neutrophil **(B)**, monocyte **(C)**, lymphocyte **(D)** and eosinophil **(E)** numbers 7 days after infection. Data (mean ± SEM) was analyzed by Student t test. *P < 0.05, **P < 0.01.
