# Supplementary figures and images for: Myeloid Protease-Activated Receptor-2 Contributes to Influenza A Virus Pathology in Mice
Source: Front Immunol. 2021 Dec 1;12:791017. doi: 10.3389/fimmu.2021.791017 (PMC8671937; doi:10.3389/fimmu.2021.791017)

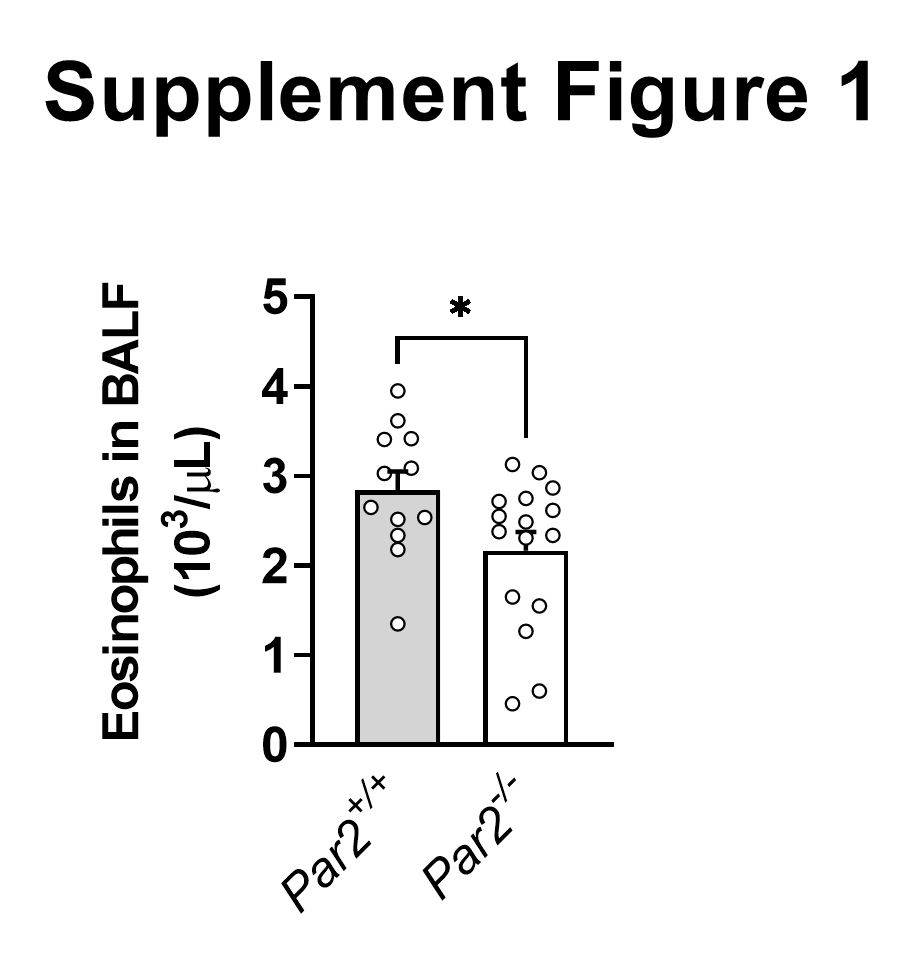

Supplement: Supplement Figure 1 — PAR2 deficiency was associated with reduced eosinophil numbers in the airspace 3 days after IAV infection. Par2 +/+ and Par2 -/- mice were infected with 0.04 HAU IAV and eosinophil numbers in bronchoalveolar lavage fluid (BALF) was analyzed by automated cell counter 3 days after infection. Data (mean ± SEM) was analyzed by Student t test. *P < 0.05. [file Image_1.tif]

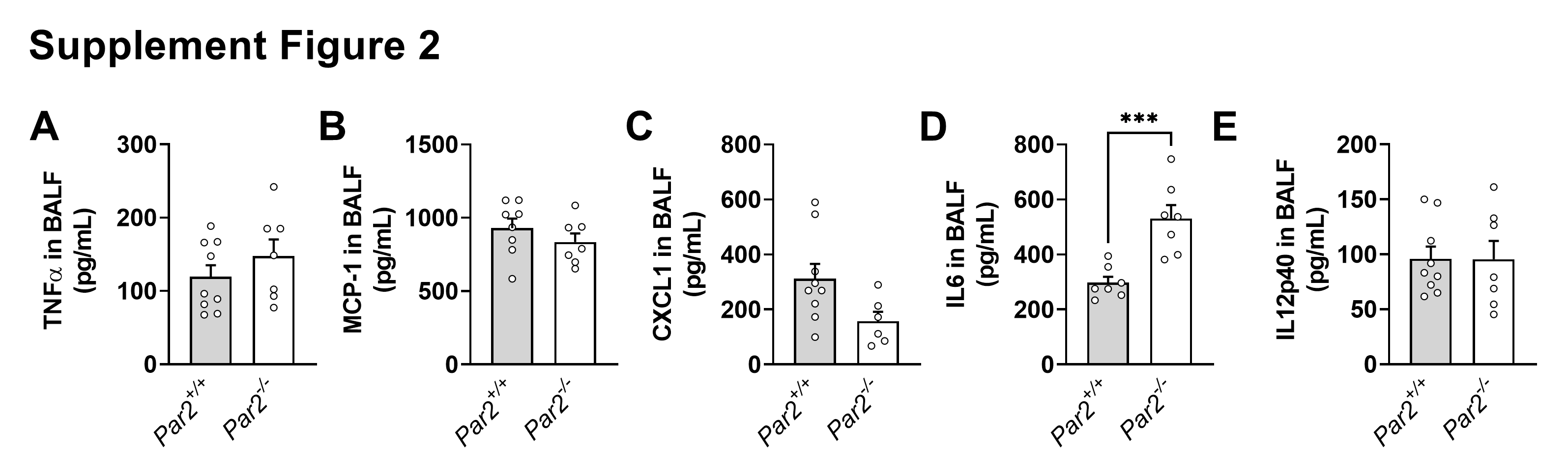

Supplement: Supplement Figure 2 — Cytokine levels in airspace of Par2 +/+ and Par2 -/- mice 7 days after influenza A virus infection. Par2 +/+ and Par2 -/- mice were infected with 0.04 HAU IAV and bronchoalveolar lavage fluid (BALF) was analyzed for TNFα (A), MCP-1 (B), CXCL1 (C), IL6 (D) and IL12p40 (E) protein levels 7 days after infection by ELISA. Data (mean ± SEM) was analyzed by Student t test. ***P < 0.005. [file Image_2.tif]

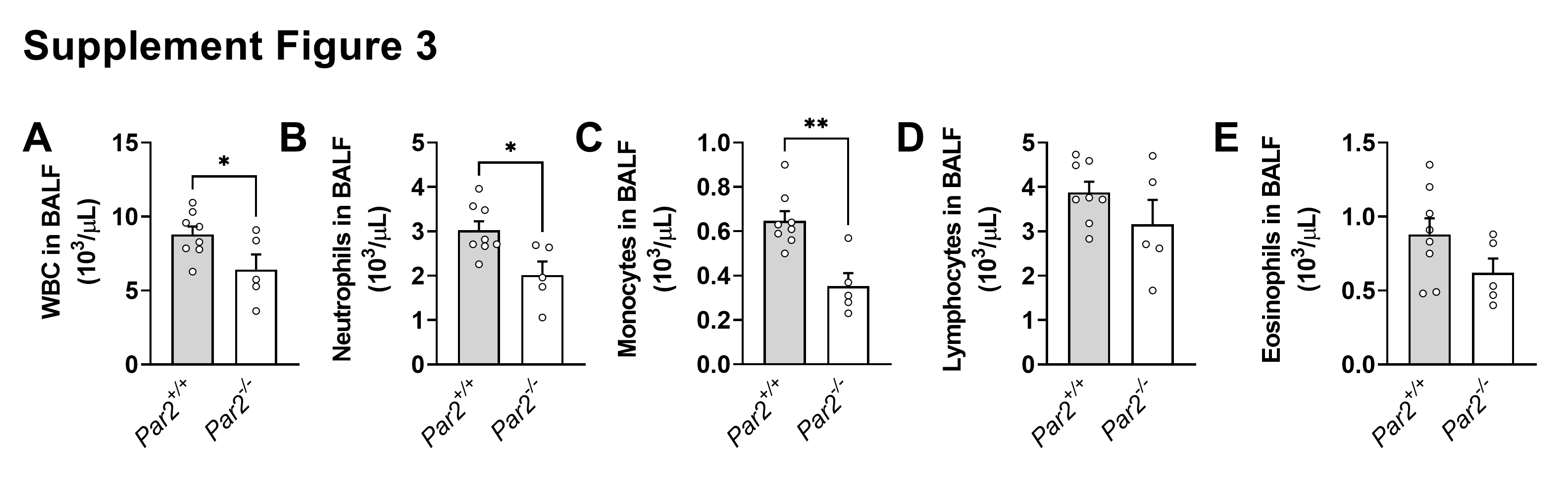

Supplement: Supplement Figure 3 — PAR2 deficiency was associated with reduced immune cell numbers in the airspace 7 days after influenza A virus infection. Par2 +/+ and Par2 -/- mice were infected with 0.04 HAU IAV and bronchoalveolar lavage fluid (BALF) cellularity was analyzed by automated cell counter for total white blood cell (WBC) (A), neutrophil (B), monocyte (C), lymphocyte (D) and eosinophil (E) numbers 7 days after infection. Data (mean ± SEM) was analyzed by Student t test. *P < 0.05, **P < 0.01. [file Image_3.tif]
